# Ferroptosis: A potential opportunity for intervention of pre-metastatic niche

**DOI:** 10.3389/fonc.2022.980620

**Published:** 2022-09-09

**Authors:** Shenghua Zhuo, Liangwang Yang, Shenbo Chen, Caiying Tang, Weicheng Li, Zhenzhong Gao, Jigao Feng, Kun Yang

**Affiliations:** ^1^ Department of Neurosurgery, First Affiliated Hospital of Hainan Medical University, Haikou, China; ^2^ Department of Neurosurgery, Second Affiliated Hospital of Hainan Medical University, Haikou, China

**Keywords:** ferroptosis, pre-metastatic niche, tumor, immune escape, therapeutic strategies

## Abstract

It is widely thought that the tumor microenvironment (TME) provides the “soil” for malignant tumors to survive. Prior to metastasis, the interaction at the host site between factors secreted by primary tumors, bone-marrow-derived cells, with stromal components initiates and establishes a pre-metastatic niche (PMN) characterized by immunosuppression, inflammation, angiogenesis and vascular permeability, as well as lymphangiogenesis, reprogramming and organotropism. Ferroptosis is a non-apoptotic cell death characterized by iron-dependent lipid peroxidation and metabolic constraints. Ferroptotic cancer cells release various signal molecules into the TME to either suppress or promote tumor progression. This review highlights the important role played by ferroptosis in PMN, focusing on the relationship between ferroptosis and PMN characteristics, and discusses future research directions.

## Introduction

It is well-established that tumor cells spread from the primary to other single or multiple sites nearby or far away *via* blood vessels and lymphatic vessels by separation, migration, invasion and adhesion (1). Notably, tumor metastasis is difficult to cure once it occurs. Metastasis accounts for about 90% of all cancer-related mortality (2). Therefore, early detection, accurate diagnosis and personalized treatment strategies before the onset of metastasis are warranted to cure malignant tumors.

As early as 1889, British surgeon Stephen Paget put forward the “seed” and “soil” hypothesis, whereby cancer cells are analogous to the “seed” and metastatic sites to the “soil” (3). As the “soil” of tumor metastasis, the complex tumor microenvironment (TME) at the metastatic site is closely related to the malignant behavior of tumor cells. Primary tumor cells can create a microenvironment that promotes colonization and growth before metastasis. Prior to metastasis, the metastatic site forms a pre-metastatic niche (PMN) with immunosuppression, inflammation, angiogenesis and vascular permeability, as well as lymphangiogenesis, reprogramming and organotropism characteristics. Functionally, the PMN promotes tumor cell metastasis through four stages, namely priming, licensing, initiation, and progression, to facilitate tumor cell colonization and growth (4). During this spatial time course, tumor cells recruit tumor-mobilized bone marrow-derived cells and various inhibitory and regulatory immune cells by secreting multiple tumor-derived secreted factors (TDSFs) and extracellular vesicles (EVs). Moreover, they interact with the host matrix to form PMN characterized by immunosuppression and extracellular matrix (ECM) remodeling, a phenomenon that enables circulating tumor cells (CTCs) to not only evade the body’s surveillance and pursuit but also take root, sprout and grow in the “fertile soil” (5).

In 2012, researchers discovered that certain lethal compounds could induce a new mode of cell death. This type of cell death, termed ferroptosis, manifests as iron-dependent and reactive oxygen species (ROS) accumulation and is morphologically, genetically, and biochemically distinct from necrosis, apoptosis, and autophagy (6). An increasing body of evidence suggests that ferroptosis is not only closely linked to cancer-acquired drug resistance and immune evasion but also has dual effects (7, 8).

Epithelial-to-mesenchymal transition (EMT) in tumors refers to the dynamic changes in cellular organization from an epithelial to a mesenchymal phenotype, which cause functional changes in cell migration and invasion. Previous studies have shown that EMT plays an irreplaceable role in tumor metastasis (2), can also lead to metastatic spreading of tumors and confer drug resistance to clinical treatment (9). Interestingly, EMT signaling is closely related to ferroptosis (7). Ferroptosis induced *via* inhibition of the lipid peroxidase signaling pathway is a feature in therapy-resistant cancer cells of various mesenchymal cell states (10). This pathway, which centers around the metabolism of long-chain polyunsaturated fatty acids (PUFAs), is regulated by the EMT-inducible transcription factor zinc finger E-box homeobox 1 (ZEB1). Moreover, glutathione-dependent antioxidant enzyme glutathione peroxidase 4 (GPX4) dissipates reactive peroxides produced by the metabolism of PUFAs and prevents ferroptotic cell death (10). Combination treatment, comprising the ferroptosis inducers (FINs) beta-elemene and cetuximab have been reported to sensitize KRAS-mutated colorectal cancer cells by inhibiting EMT and lymph node (LN) metastasis and induce ferroptosis (11). Furthermore, epigenetic reprogramming of EMT is associated with ferroptosis (12). Although the role of ferroptosis in PMN is currently unclear, there appears to be a link between ferroptosis and PMN. This review focuses on the relationship between ferroptosis and PMN characteristics (including immunosuppression, inflammation, angiogenesis and vascular permeability, as well as lymphangiogenesis, reprogramming and organotropism), and highlights potential areas and directions for future research.

## Immunosuppression

Cancer cells metastasize by evading immune surveillance. The primary tumor either forms TME or PMN through recruitment and expansion of immunosuppressive cell populations. It also transforms the phenotype and function of normal immune cells, from a potential immune response to a tumor-promoting state. Notably, tumor-promoting immune cells not only enhance immune evasion by suppressing antitumor immune responses, but also promote tumor cell invasion by inducing an immunosuppressive microenvironment, promoting EMT, and inducing vasculature at the primary tumor or metastatic site to establish PMN. Collectively, these phenomena eventually lead to tumor metastasis (13).

Cell death can be divided into two types, namely tolerogenic cell death (TCD) and immunogenic cell death (ICD), depending on whether an immune response is induced. In TCD, phagocytes efficiently eliminate cellular debris, *via* endocytosis, critical for preventing inflammatory and autoimmune diseases. ICD entails the release of intracellular molecules or they are exposed to dead or dying cells, a phenomenon that elicits adaptive immunity that subsequently induces immune responses to tumor-associated antigens (14). Among them, damage-associated molecular patterns (DAMPs) and cytokines released during cell death are critical for initiating immune responses (15, 16). The high mobility group box 1 protein (HMGB1), an essential protein required for cancer cell immunogenicity, is a crucial DAMP that promotes antigen presentation by dendritic cells (DCs) to T cells (17). Cancer cells with compound-induced ferroptosis were found to release HMGB1 in an autophagy-dependent manner (18). Remarkably, ferroptosis was recently identified as a type of ICD. Additional evidence showed that the GPX4 inhibitor RSL3 induced early ferroptotic cancer cells was ICD and was accompanied by the production of DAMPs, such as purine adenosine triphosphate (ATP) and HMGB1. Functionally, these components not only trigger *in vitro* maturation of DCs but also elicit vaccination-like effects in immunocompetent mice (19). In breast cancer, patrolling monocytes establish early interactions with metastatic tumor cells, clear tumor material in blood vessels, and promote recruitment as well as activation of natural killer cells (NK cells), thereby inhibiting tumor cells metastasis (20, 21). Studies have associated hypoxic features in the TME with PMN formation, while TDSFs generated by hypoxia in primary tumors can recruit immunosuppressive cells and reduce NK cells cytotoxicity in PMN (22). Peripheral blood mononuclear cells (PBMCs) have the potential to differentiate into various types of immune cells, under physiological and pathological conditions, while erastin (a FIN) plays an immunomodulatory function, where it promotes proliferation and differentiation of human PBMCs into B and NK cells (23). Another study showed that interferon γ (IFNγ), which is mainly produced by T lymphocytes or NK cells, sensitizes hepatocellular carcinoma cells to ferroptosis by activating the JAK/STAT signaling inhibitory system xc-activation, and promoting lipid peroxidation associated with mitochondrial damage (24). Interestingly, activation of CD8^+^ T cell activation has been documented to induce ferroptosis in cancer cells. Notably, activation of CD8^+^ T cells by nivolumab-based immunotherapy mediated downregulation of solute carrier family 3 membrane 2 (SLC3A2) and solute carrier family 7 membrane 11 (SLC7A11) by releasing IFNγ. This phenomenon inhibited uptake of cystine by tumor cells and promoted occurrence of lipid peroxidation and ferroptosis in cancer cells (25). IFNγ from CD8^+^ T cells and arachidonic acid (AA) from the tumor microenvironment mediate tumorigenic ferroptosis through acyl-CoA synthetase long-chain family member 4 (ACSL4) while targeting tumor ACSL4 reportedly improved immune checkpoint blockade treatment sensitivity (26). On the other hand, exposure to immune-activated cells to ferroptosis may impair their antitumor ability. Notably, conditional deletion of Gpx4 induced ferroptosis by lipid peroxidation in mouse T cells (27), while CD36 reportedly mediated AA uptake by CD8^+^ T cells, thereby causing lipid peroxidation and ferroptosis, as well as impairing the antitumor function of CD8^+^ T cells (28). DCs selectively induced by RSL3 undergo ferroptosis and cannot induce CD8^+^ T cells to produce IFNγ (29). Impaired toxicity of NK cells isolated from the ovarian cancer microenvironment and peripheral blood NK cells exposed to the tumor-derived ascites microenvironment showed cell morphology consistent with ferroptosis (30). Antitumor activity of NK cells could be restored by activation of the nuclear factor E2-related factor 2 (NRF2) antioxidant pathway (30, 31).

There is ample evidence that myeloid-derived suppressor cells (MDSCs) and tumor-associated macrophages (TAMs) suppress antitumor immune responses in PMN (32, 33). MDSCs can be induced to differentiate into TAMs under inflammatory stress and hypoxic niche (33). As optimal partners of tumor cells, MDSCs are a population of immature myeloid cells that emerge and accumulate in PMN, where they support tumor cell colonization into PMN during metastasis (34). Induction of ferroptosis in MDSCs may be an effective therapy to inhibit the accumulation of MDSCs during cancer immunotherapy (35). The chemotherapeutic drug gemcitabine (Gem), a FIN, can effectively deplete MDSCs. For instance, a previous study showed that Gem nanoparticles could promote anti-melanoma immunity, effectively deplete MDSCs and regulatory T cells (Tregs), and polarize TAMs to antitumor M1 phenotype, and also improve CD8^+^ T cell immune response, thereby inhibiting tumor growth (36). Furthermore, activating transcription factor 4 (ATF4) reportedly upregulated expression of heat-shock 70kDa protein 5 (HSPA5) in pancreatic ductal adenocarcinoma (37). HSPA5 binds to GPX4 and avoids GPX4 protein degradation and lipid peroxidation, thus limiting Gem’s anticancer activity. However, promoting ferroptosis was found to enhance Gem sensitivity (37). Similarly, combining FINs and apoptosis activators was shown to significantly improve the cytotoxic effect of Gem in pancreatic cancer (38). Notably, N-acylsphingosine amidohydrolase 2 (ASAH2) suppresses the p53 pathway to protect MDSCs from ferroptosis by destabilizing p53 protein in TME (35).

**Figure 1:**
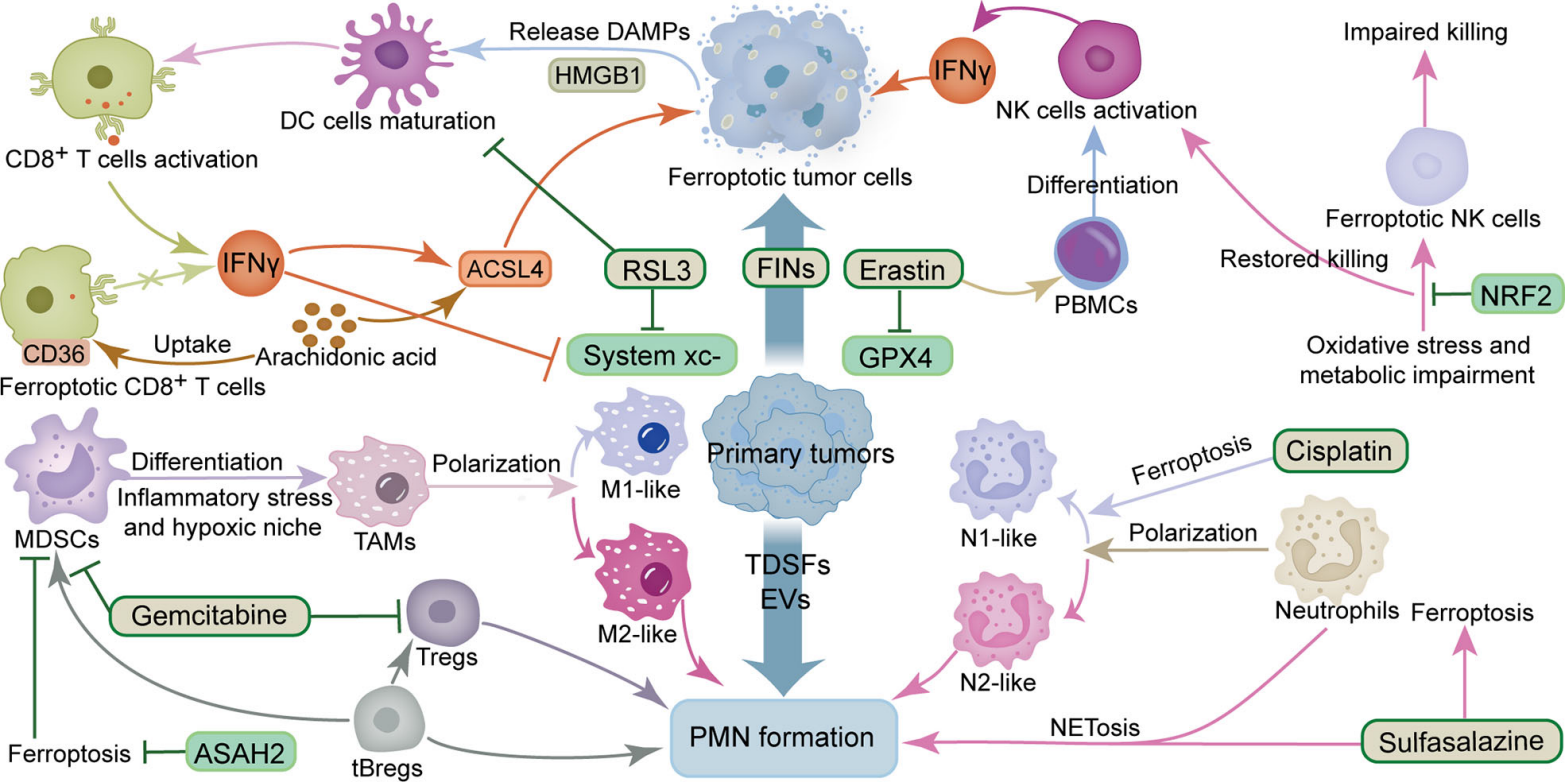
Crosstalk between ferroptosis and immunosuppression and inflammation in PMN. The ingredients that promote the formation of PMN include TDSFs, EVs, MDSCs, M2-like TAMs, tBregs, Tregs and N2-like neutrophils. The components that inhibit the formation of PMN include IFNγ, DC cells, NK cells, M1-like TAMs, N1-like neutrophils and CD8^+^ T cells. The ingredients that promote ferroptosis include RSL3, erastin, gemcitabine, sulfasalazine, cisplatin, IFNγ, ACSL4 and arachidonic acid. The components that inhibit ferroptosis include GPX4 and system xc-. ACSL4, acyl-CoA synthetase long-chain family member 4; ASAH2, N-acylsphingosine amidohydrolase 2; DAMPs, damage-associated molecular patterns; DC cells, dendritic cells; EVs, extracellular vesicles; FINs, ferroptosis inducers; GPX4, glutathione peroxidase 4; HMGB1, high mobility group box 1 protein; IFNγ, interferon γ; MDSCs, myeloid-derived suppressor cells; NK cells, natural killer cells; NRF2, nuclear factor E2-related factor 2; PBMCs, Peripheral blood mononuclear cells; PMN, pre-metastatic niche; TAMs, tumor-associated macrophages; tBregs, tumor-evoked regulatory B cells; TDSFs, tumor-derived secreted factors; Tregs, regulatory T cells.

Macrophages are a vital factor in the progression of cancer metastasis, owing to their effect on PMN formation and CTCs adhesion, as well as extravasation, and colonization (39). Studies have shown that tumor-derived exosomes promote tumor metastasis by stimulating macrophage development in PMN to an immunosuppressive phenotype through glycolytic-dominant metabolic reprogramming (40). ICD induced triggered by FINs has been reported to polarize TAMs to a pro-immune antitumor phenotype, thereby enabling ferroptosis and immune regulation to act synergistically (41, 42). Additional evidence has shown that macrophages can efficiently clear ferroptotic cancer cells by phagocytosis (43), while ferric citrate effectively induces ferroptosis in macrophages (44). Furthermore, different subsets of macrophages have various sensitivities to ferroptosis. Particularly, the M1 type exhibits higher levels of resistance to drug-induced ferroptosis compares to the M2 type, while tumor suppressor M1-type macrophages are more resistant to FINs than tumor-promoting M2-type macrophages. This phenomenon may be attributed to the higher inducible nitric oxide synthase (iNOS) content in M1 than M2 macrophages. The lower iNOS content makes M2 macrophages produce less NO free radicals, a phenomenon that reduces their inhibitory effect on lipid peroxidation, thus rendering them sensitive to ferroptosis (45). Interestingly, different B cell subsets also differ in their sensitivity to ferroptosis. For instance, B1 and marginal zone B cells display higher lipid metabolism and are more sensitive to lipid peroxidation as well as ferroptosis compared to follicular B2 counterparts (46). Tumor-evoked regulatory B cells (tBregs), which are phenotypically similar to activated but less proliferative mature B2 cells, reportedly promote breast cancer metastasis by converting quiescent CD4^+^ T cells into Tregs (47). Moreover, tBregs can also directly activate the regulatory functions of MDSCs-derived monocytes and granulocyte subsets, while activated MDSCs have been shown to improve production of ROS and NO, and more effectively inhibit CD4^+^ T and CD8^+^ T cells, thereby promoting tumor metastasis (48). Tregs activation and suppression of antitumor immunity are maintained by GPX4 by preventing lipid peroxidation and ferroptosis (49). Taken together, findings from studies demonstrate that ferroptosis is closely associated with various immune factors in PMN, especially the complex crosstalk between ferroptosis and tumor cells and immune cells. The sensitivity of different immune cells and tumor types to FINs appears to determine the fate of PMN formation; nonetheless, further research explorations are needed to answer this question (**Figure 1**).

## Inflammation

Overwhelming evidence substantiates that chronic inflammation transforms susceptible cells into tumors, subsequently driving tumor development and metastasis (50, 51). Establishing an inflammatory microenvironment at the host site facilitates the seeding, survival and proliferation of CTCs in PMN (4). In addition, balancing ROS production and antioxidant defenses is critical for tumor cell survival and growth, owing to the fact that high ROS levels cause cytotoxicity. In contrast, low or moderate ROS levels have been implicated in DNA damage and mutation, as well as development of inflammatory responses and ultimately carcinogenesis (52). Accordingly, ferroptosis plays an essential role in the inflammatory process, representing a new therapeutic target in many inflammatory diseases (53, 54).

The crosstalk between chronic inflammation and various immune and inflammatory cells promotes tumor cell metastasis (55). In peri-tumor-associated inflammatory cells, GPX4 deletion in myeloid cells mediated an increase in ROS production, which was accompanied by secretion of excess H_2_O_2_, transforming intestinal epithelial cells (IECs) by triggering DNA mutations (56). H_2_O_2_ also induces secretion of chemokines and cytokines by IECs through the tumor necrosis factor α (TNFα) autocrine loop to recruit myeloid cells and promote tumor invasion (56). Neutrophils in TME can display opposing phenotypes: tumor-fighting effector cells (N1) and tumor-promoting effector cells (N2). Studies have shown that neutrophil recruitment is critical for PMN formation. In fact, neutrophils can spontaneously migrate during tumor progression (57). Chronic nicotine exposure has been shown to recruit tumor-promoting N2-type neutrophils, induce pulmonary PMN formation, and promote tumor cell colonization as well as metastatic growth (58). A previous study found that primary tumor-derived exosomes could activate lung epithelial cell Toll-like receptor 3 (TLR3) to initiate neutrophil recruitment and pulmonary PMN formation (59). An antitumor effect was observed in N1-polarized neutrophils induced by cisplatin-mediated non-small cell lung cancer ferroptosis (60). As found in another study, platinum prodrug nanoparticles induced ferroptosis by concurrent chemoradiotherapy to inhibit tumor recurrence and metastasis (61). Moreover, neutrophil infiltration in glioblastoma necrotic sites induces ferroptosis (62). There is ample evidence suggesting that sulfasalazine, an inhibitor of system xc-, can induce tumor ferroptosis (63, 64). Interestingly, sulfasalazine-induced accumulation of oxidized phospholipids in activated neutrophils could promote neutrophil extracellular traps-induced specific death mode NETosis (65, 66). Notably, NETosis is well-established to promote tumor growth and metastasis (67–69), suggesting that complex linkages between cell death pathways influence disease progression. Given the complex crosstalk between different forms of cell death (70), it is essential to fully understand and elucidate which form of cell death dominates in a given physiological or pathological setting.

Pro-inflammatory cytokines secreted by tumor cells or stromal cells, such as interleukin 6 (IL6), IL1β and C-C motif chemokine ligand 2 (CCL2), can induce phenotypic changes in tumor cells, recruit bone marrow-derived cells and form an inflammatory environment. Collectively, they constitute PMN and facilitate colonization of metastatic cells (71). Studies have shown that chronic inflammatory cytokines can also work synergistically with immunosuppressive cells, such as TAMs, to build an immunosuppressive microenvironment (72). Ferroptotic cells have been reported to induce aggregation and chemotaxis of macrophages through CCL2 and secrete cytokines, such as IL6 and IL1β, thereby triggering inflammation and enhancing tissue damage (73). In addition, ferroptosis-related genes play a crucial role in formation of inflammation, and ruptured ferroptotic cells can cause necrotizing inflammation and release pro-inflammatory DAMPs. Moreover, GPX4 controls lipoxygenase (LOX) and prostaglandin internalization through peroxide elasticity activity of prostaglandin-endoperoxide synthase enzymes involved in ferroptosis at multiple levels (74). As DAMPs, modulating HMGB1 activity in inflammation, immune response and tissue repair could generate invaluable treatment strategies for various diseases, including cancer (75). A previous study found that LPS could induce acetylation of HMGB1 in colon cancer cells, thereby promoting its interaction with GPX4, and regulating ROS levels and inflammation (76).

Macrophages are “gatekeepers” whose diversity and heterogeneity are critical in maintaining iron homeostasis (77). M1 macrophages, also known as inflammatory macrophages, can be activated by IFNγ, TNFα, HMGB1 and lipopolysaccharide (LPS), whereas M2 macrophages, also known as anti-inflammatory macrophages, can be activated by IL4, IL10, IL13, colony-stimulating factor 1 (CSF1) and transforming growth factor β (TGFβ) (78). Both have different phenotypes and functions (33). In contrast to the iron-chelating phenotype of inflammatory macrophages induced by pro-inflammatory cytokines and DAMPs, anti-inflammatory macrophages and lymphocytes exhibit an iron-releasing phenotype that not only contributes but also distributes iron in the TME (79). In lung cancer, iron-loaded TAMs enhance ROS production and pro-inflammatory cytokines (TNFα and IL6) to induce tumor cell death (80). Macrophage polarization is highly plastic, and its phenotype is primarily influenced by its current microenvironment, independent of the previous polarization state (81). TAMs actively release iron to the TME in hyper-inflamed tumors, thereby enriching the TME with iron and promoting both cancer development and immune escape. Notably, excess iron predisposes macrophages to a pro-inflammatory phenotype (79). Interestingly, M2 macrophages are more sensitive to ferroptosis (45). Therefore, harnessing the selective sensitization of macrophages to ferroptosis may improve a tumor’s inflammatory microenvironment. Given the complex crosstalk between ferroptosis and iron metabolism with tumor inflammation and immune cells, it is critical to investigate the impact of immune cell heterogeneity and plasticity, such as TAMs, on iron homeostasis as well as the balance in the inflammatory microenvironment. Inducing iron overload to promote ferroptosis and remodel the inflammatory microenvironment represents a potential therapeutic strategy to improve PMN formation (**Figure 1**).

## Angiogenesis and vascular permeability

**Figure 2:**
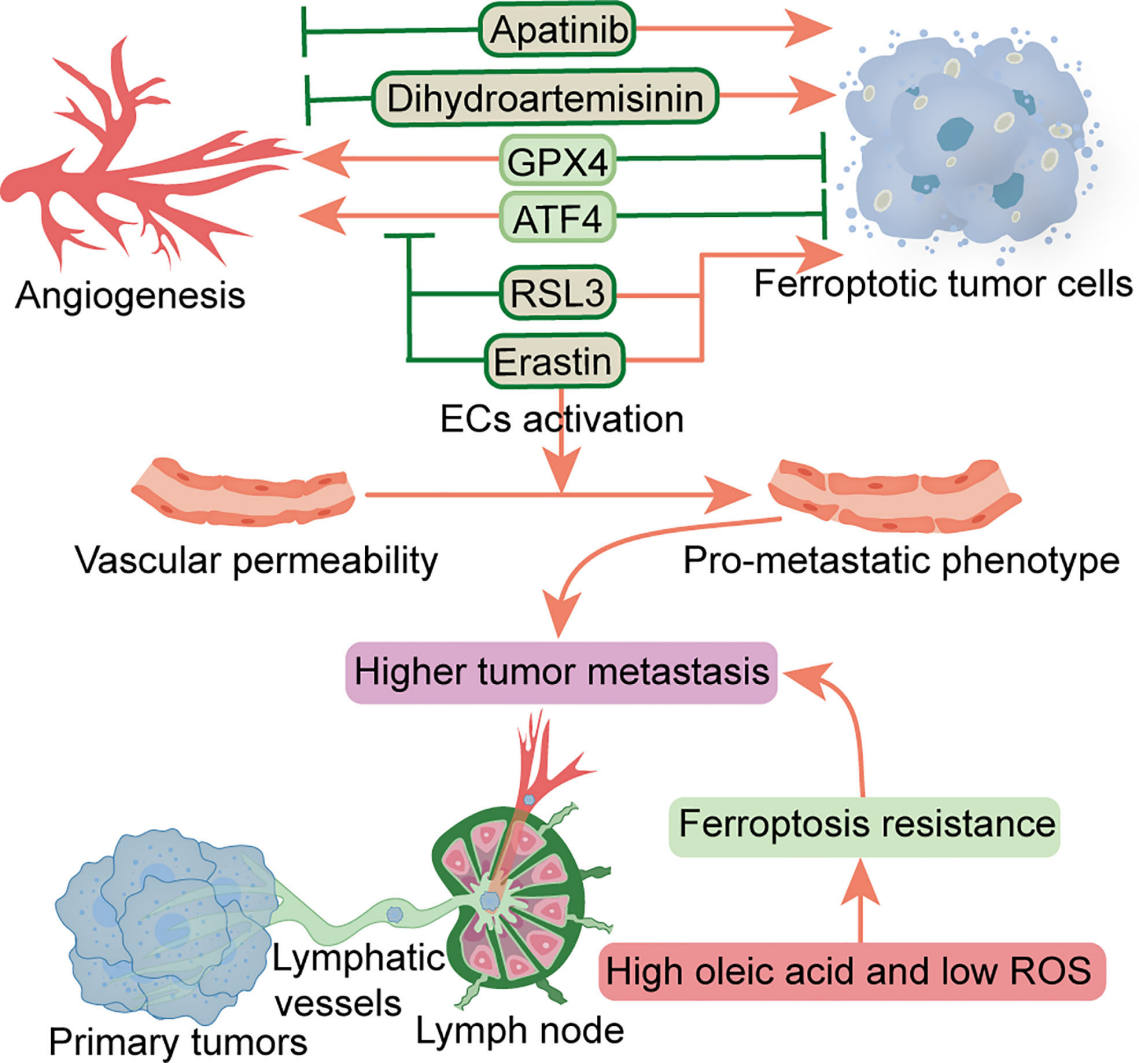
Relationship between ferroptosis and angiogenesis and vascular permeability and lymphangiogenesis. Factors that inhibit angiogenesis and promote ferroptosis include RSL3, erastin, dihydroartemisinin and apatinib. Erastin can also promote vascular permeability. ATF4 and GPX4 can promote angiogenesis and inhibit ferroptosis. Metastatic tumor cells escape ferroptosis through lymphatic vessels in low ROS and high oleic acid lymph node microenvironment. ATF4, transcription factor 4; ECs, endothelial cells; GPX4, glutathione peroxidase 4; ROS, reactive oxygen species.

Multiple factors have been shown to affect angiogenesis in the TME, including various peritumoral cells, ECM, tumor metabolism, and tumor-derived extracellular vesicles (82). Increased angiogenesis and permeability in PMN can reportedly promote metastasis (4). At the pre-metastatic stage, primary tumors upregulate angiopoietin 2 (ANGPT2), matrix metalloproteinase 3 (MMP3) and MMP10 in the lung, increasing pulmonary vascular permeability and extravasation of CTCs, thereby promoting lung metastasis (83). A previous study found that TAMs-derived vascular endothelial growth factor (VEGF) improved vascular permeability, thereby promoting cancer cell invasion and metastasis (84). Another study showed that cancer-derived exosomal miR-25-3p participates in PMN formation and promotes colorectal cancer metastasis by inducing angiogenesis and permeability (85). Notably, ferroptosis has been closely associated with regulation of angiogenesis and permeability. As a crucial regulator of angiogenesis, ATF4 overexpression reportedly conferred resistance to sorafenib and erastin in glioma cells. Moreover, ATF4-induced angiogenesis was effectively inhibited by erastin and RSL3 (86), while another study demonstrated that GPX4 acts as an essential regulator of tumor angiogenesis and vascular maturation by controlling the activity of 12/15-LOX (87). Dysregulation of glutamate transport can enhance the function of Tregs and promote anti-VEGF treatment resistance in glioblastoma. Notably, blocking and eliminating Tregs using CD25 before anti-VEGF treatment reportedly restored production of IFNγ by CD8^+^ T cells, thereby improving the antitumor response of anti-VEGF therapy (88). Dihydroartemisinin, a semi-synthetic derivative of artemisinin, has been reported to exhibit antitumor activity in head and neck cancer cells by inhibiting angiogenesis, and also inducing ferroptosis and apoptosis (89). Apatinib, an anti-angiogenic drug for the treatment of metastatic gastric cancer, is a highly selective inhibitor of VEGFR2 that induces ferroptosis by reducing cellular glutathione levels and increasing levels of lipid peroxidation (90). Conversely, the application of erastin reportedly activated vascular endothelial cells to a leakier pro-metastatic phenotype, thereby promoting breast cancer cell adhesion and transendothelial migration (91). As the main characteristics of malignant tumor PMN, angiogenesis and vascular permeability represent essential steps that precede metastasis. Although FINs can inhibit angiogenesis, FINs may also increase the permeability of vascular endothelial cells, thereby triggering entry of tumor cells into blood vessels to facilitate hematogenous metastasis. To date, however, it is not known whether ferroptosis can alter this characteristic and interfere with PMN formation, necessitating further research explorations. In addition, the effects of FINs on this characteristic may be heterogeneous in different malignancies (**Figure 2**).

## Lymphangiogenesis

Tertiary lymphoid structures, composed of LNs and lymphatic vessels, are involved in PMN formation and tumor metastasis (92). Before metastatic spread, the primary tumor promotes lymphangiogenesis and high endothelial venule remodeling by secreting soluble factors or releasing EVs transported by lymphatic vessels, forming PMN in the LNs, leading to subsequent survival and growth of metastatic cancer cells (93). It has been established that lymphangiogenic growth factors VEGFC and VEGFD, secreted by a primary tumor, can promote lymphatic metastasis (94). VEGFD can down-regulate the 15-hydroxyprostaglandin dehydrogenase (HPGD) that degrades prostaglandins, and cause secretion of a large number of prostaglandins from the collecting lymphatic endothelial cells. Consequently, this promotes the entry of tumor cells into primary lymphatic vessels and induces collecting lymphatic vessels’ expansion to facilitate the transfer of tumor cells to distant metastases (94). Additional studies have shown that specific lymphoid tissue and immune response signatures contribute to PMN formation in sentinel LN in early-stage cervical cancer (95), while fatty acid oxidation (FAO) is a driver of LN metastasis (96). It is well-recognized that the LN microenvironment is rich in lipids, while fatty acids are a preferential energy source for LN metastatic tumor cells. Notably, LN metastasis requires tumor cells to undergo a metabolic transition to FAO (97). In pre-metastatic LN, melanoma-derived EVs fuse with subcapsular sinus CD169^+^ macrophages to avoid immune recognition, while the destruction of subcapsular sinus CD169^+^ macrophages allows EVs to enter the LN cortex to interact with B cells and activate pro-inflammatory cells tumor B cell immunity (98). Communication between EVs and LN was found to contribute to PMN formation and suppression of tumor immunity (99). Interestingly, melanoma cells experience less oxidative stress, owing to higher glutathione and oleic acid levels coupled with less free iron in the lymphatic microenvironment. Among them, tumor cells are protected from ferroptosis, a phenomenon that improves the ability to form metastatic tumors (100) (**Figure 2**). Additionally, as a vital feature of TME, the hypoxic environment affects the phenotype of various types of cells around the tumor cells (101). Moreover, angiogenesis and lymphangiogenesis are dependent on a hypoxic environment (102), while hypoxia reportedly improves resistance of malignant mesothelioma cells to ferroptosis (103). Similarly, studies have also shown that mitochondrial ferritin (FT) and FT heavy chain synergistically protect macrophages from RSL3-induced ferroptosis under hypoxic conditions (104). Therefore, the role of ferroptosis in angiogenesis and vascular permeability, as well as lymphangiogenesis in PMN warrants further study in the hypoxic environment. Future studies are expected to determine whether induction of ferroptosis can potentiate efficacy of anti-angiogenic or lymphangiogenic therapy for inhibition of tumor metastasis.

## Reprogramming

Stromal and metabolic reprogramming has been associated with PMN-promoted tumor metastasis (4). Notably, tumor stroma not only promotes cancer growth, survival, and invasion but also modifies the behavior of stromal cells, including fibroblasts and immune cells, to drive metastasis and resist therapy (105). As an important part of TME stromal cells, cancer-associated fibroblasts (CAFs) are the “architects” of matrix remodeling and are closely related to the poor prognosis of solid tumors (106). Previous studies have shown that CAFs can interact with tumor and immune cells by releasing various regulatory factors, synthesizing and remodeling the ECM, as well as inducing EMT and drug resistance (107). CAF-derived EVs reportedly induced lung PMN formation in mice, thereby improving the lung metastatic ability of salivary gland adenoid cystic carcinoma (108). Disseminated breast cancer cells induce an inflammatory phenotype in lung fibroblasts, thereby creating a microenvironment that supports metastasis (109). Another study found that CAF-derived exosomal miR-522 not only inhibited ferroptosis but also promoted acquired cisplatin and paclitaxel resistance in gastric cancer (110). Collectively, these studies substantiate that CAFs and PMN formation is closely associated with ferroptosis, suggesting the importance of CAFs in matrix reprogramming. It is unknown whether CAFs undergo ferroptosis and inhibit PMN formation, nor is it clear whether targeting CAFs can enhance the sensitivity of tumor cells to ferroptosis. Accordingly, further research is essential to elucidate these questions.

**Figure 3:**
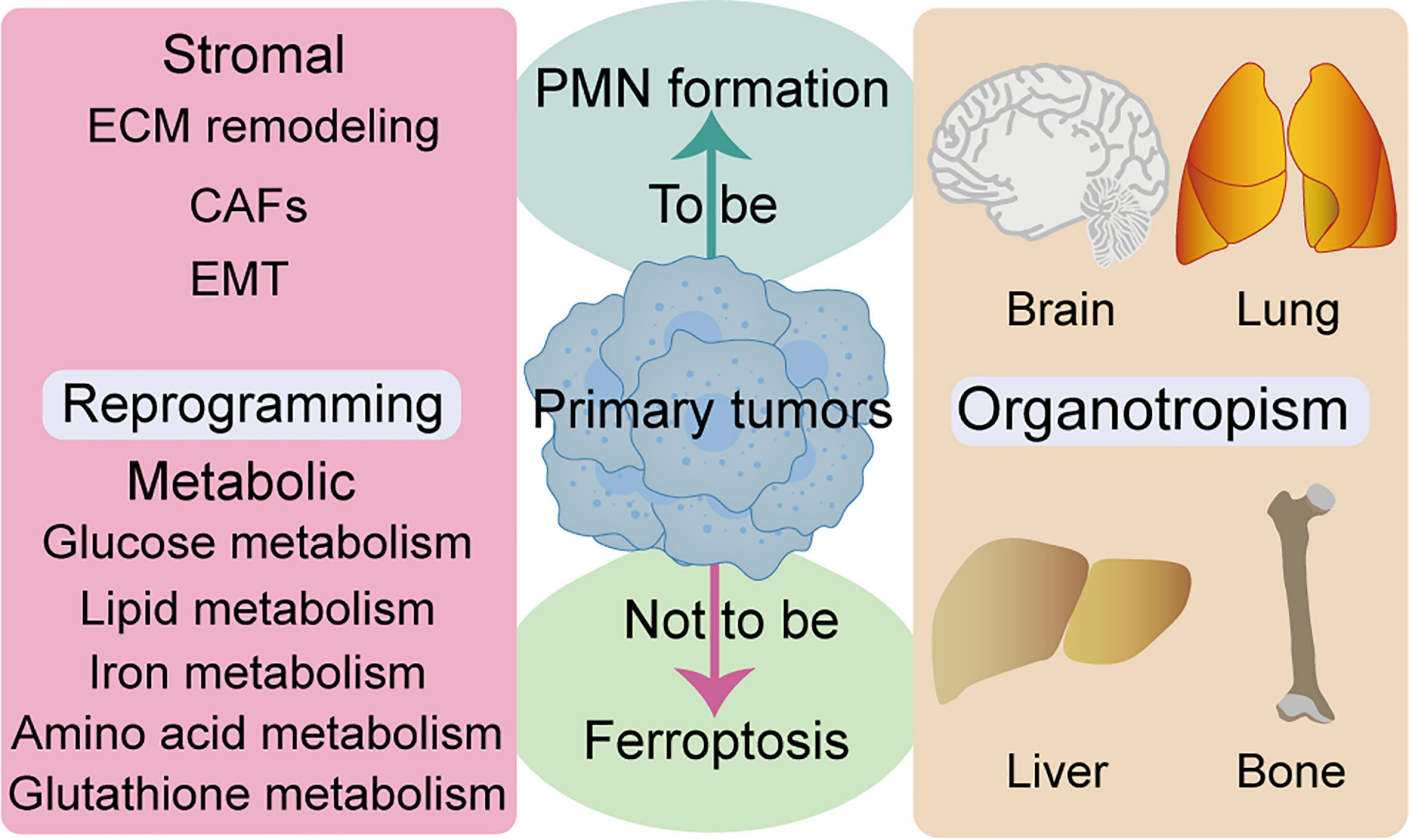
Relationship between ferroptosis and reprogramming and organotropism. ECM remodeling, CAFs, and EMT are closely associated to stromal reprogramming and ferroptosis. Multiple metabolic pathways are closely related to ferroptosis, such as glucose metabolism, lipid metabolism, iron metabolism, amino acid and glutathione metabolism. Several organs are the host parts of metastasis and are prone to ferroptosis, such as brain, lung, liver and bone. CAFs, cancer-associated fibroblasts; ECM, extracellular matrix; EMT, epithelial-to-mesenchymal transition.

Metabolic reprogramming is a hallmark of malignancy, while metabolic properties and preferences of tumors change during cancer progression (111). Generally, metastatic cancer cells selectively and dynamically adjust their metabolism at each step in the metastatic cascade. To adapt to a new environment, for survival and growth, many metastatic tumors exhibit metabolic profiles that differ from those at the primary site (112). Several review articles have shown that metabolic pathways, such as glucose metabolism, lipid metabolism, iron metabolism, amino acid and glutathione metabolism, are closely related to ferroptosis (113–115). AMP-activated protein kinase (AMPK), a central regulator of energy homeostasis and cellular metabolism, has been shown to play a tumor-promoting or antitumor effect under different circumstances. Notably, AMPK activation reportedly endows tumor cells with a growth advantage by modulating their metabolic plasticity, thereby adapting to metabolic stress (116). Mitochondrial ROS is a physiological activator of AMPK. Notably, AMPK negative feedback limits ROS production, while mitochondrial ROS-activated AMPK can regulate the stability of hypoxia-inducible factor 1α (HIF1α) (117). Similarly, AMPK plays a dual role in regulation of ferroptosis, mainly by blocking system xc- activation (118). In contrast, energy stress-mediated AMPK activation inhibits ferroptosis (119). Moreover, ROS also plays a dual role in cancer prevention and treatment (120). The relationship between metabolic homeostasis and ferroptosis is not static, and critical metabolic regulators may play opposite roles in differential tumor progression. Complex network communication between reprogramming and ferroptosis can promote PMN formation, and how tumor cells avoid ferroptosis through reprogramming deserves further study. Given that ferroptosis is closely associated with many metabolic pathways, exploiting metabolic fragility might be critical in unraveling therapeutic loopholes for inducing ferroptosis to inhibit tumor PMN formation (**Figure 3**).

## Organotropism

Features of organotropism may be innately associated with PMN, owing to the fact that some cancer types tend to metastasize to specific organs with selective microenvironments (4). Intrinsic properties of tumors, coupled with unique characteristics of host organs, circulation patterns, and interactions between tumor cells and the host microenvironment, jointly determine organ-specific metastatic behavior. Studies have shown that major metastatic target organs, such as bone, liver, lung, and brain, among others, have their specific PMN (121) (**Figure 3**). Moreover, researchers have documented the role of ferroptosis in many cases of pathological cell death, especially in tissues such as the liver and brain (122). Notably, iron-rich tumors, such as hepatocellular carcinoma and non-small cell lung cancer, maybe particularly responsive to FINs (7). Furthermore, different ferroptosis regulators have distinct gene expression levels throughout the tumor. SLC7A11 inhibitors have been reported to be particularly effective against certain types of cancer that overexpress this target, such as esophageal cancer and glioblastoma (7). Although no studies have demonstrated the relationship between ferroptosis with organotropism during PMN formation, iron-rich organs (more nutrient-rich “soil”) may be more susceptible to PMN formation. Moreover, ferroptosis may have particular effects on prevention and treatment of organotypic metastases in certain tumors, such as breast and lung cancer brain metastases, due to tissue specificity of ferroptosis and the high sensitivity of specific types of tumors to FINs.

## Conclusions and perspectives

Formation and maturation of PMN guarantee successful extravasation, colonization and growth of metastatic tumor cells. This complex spatiotemporal process relies on interactions between tumor cells at the primary site and the colonization site microenvironment. Notably, vascular stabilization and immune regulation represent promising pathways to deconstruct the complexity of PMN (123). This review addresses the potential link between ferroptosis and PMN, harnessing our knowledge of PMN characteristics and information from published articles. Although no direct and precise links have so far been established, this information provides a new frontier for discovery of interventions in PMN formation and maturation *via* ferroptosis to overcome cancer metastasis. In the future, more in-depth studies are needed to identify and characterize the key molecules as well as mechanisms underlying ferroptosis in PMN, as such information will guide development of safe and effective anticancer therapies. This review did not cover various topics, such as tumor cell dormancy characteristics, inter-tumor and intra-tumor heterogeneity, and cancer stem cells. Indeed, epigenetic regulation plays an integral role in malignant tumor progression (124), while ferroptosis mediates various epigenetic regulators and metabolic changes. Studies have shown that epigenetic regulation of ferroptosis may serve as a novel anticancer therapeutic strategy (12, 125). Future studies, seeking to elucidate the role of ferroptosis in PMN, should focus on epigenetic regulation.

In summary, the aforementioned six PMN characteristics not only comprehensively function from different aspects to ensure colonization and metastasis of tumor cells, but also are closely related to ferroptosis, suggesting that ferroptosis may play an essential role in PMN and modulation of ferroptosis to predict or treat tumor metastasis is an attractive strategy. However, its toxic and side effects should be borne in mind, including an elevated risk of neurodegeneration and acute tissue damage, or ineffective therapeutic effects leading to tumor progression. Therefore, researchers need to conduct sufficient preclinical studies to ascertain its safety and efficacy.

Future studies are expected to not only elucidate the mechanism underlying ferroptosis in PMN but also identify and distinguish the effect of ferroptosis on different PMN characteristics during tumor metastasis. Such research endeavors are expected to employ advanced technologies, such as organoid technology, single-cell and spatial transcriptome sequencing platforms, and nanomedicine approaches, to study the effect of ferroptosis on the interaction between tumor cells and peritumoral stromal cells and immune cells, as well as the initiation of ferroptosis in PMN priming, licensing, initiation, and progression roles in various spatiotemporal stages. Findings from these explorations will provide valuable insights to guide future development and utilization of new prevention and treatment strategies against cancer metastasis.

## Author contributions

KY, JF, and ZG conceived and designed the study. SZ, LY, and SC provided equal contributions to research article writing. CT and WL revised the manuscript. The final manuscript has been read and approved by all authors.

## Funding

This work was supported by Finance science and technology project of hainan province (Grant No. ZDYF2022SHFZ088, ZDYF2019129), the National Nature Science Foundation of China (Grant No. 82060456), the Innovative Research Project of Hainan Graduate Students (Grant No. Qhyb2021-58) and project supported by Hainan Province Clinical Medical Center.

## Conflict of interest

The authors declare that the research was conducted in the absence of any commercial or financial relationships that could be construed as a potential conflict of interest.

## Publisher’s note

All claims expressed in this article are solely those of the authors and do not necessarily represent those of their affiliated organizations, or those of the publisher, the editors and the reviewers. Any product that may be evaluated in this article, or claim that may be made by its manufacturer, is not guaranteed or endorsed by the publisher.
